# Rehabilitative Ultrasound Imaging as Visual Biofeedback in Pelvic Floor Dysfunction: A Narrative Review

**DOI:** 10.3390/tomography12010010

**Published:** 2026-01-15

**Authors:** Dana Sandra Daniel, Mila Goldenberg, Leonid Kalichman

**Affiliations:** Department of Physical Therapy, Recanati School for Community Health Professions, Faculty of Health Sciences, Ben Gurion University of the Negev, Beer Sheva 84105, Israel; da_daniel@rambam.health.gov.il (D.S.D.); milagold@bgu.ac.il (M.G.)

**Keywords:** pelvic floor dysfunction, rehabilitative ultrasound imaging, visual biofeedback, pelvic floor muscle training, physical therapy

## Abstract

*Background*: Pelvic floor dysfunction, more prevalent in women but affecting both genders, impairs sphincter control and sexual health, and causes pelvic pain. Pelvic floor muscle (PFM) training is the first-line treatment for urinary incontinence, supported by robust evidence. Rehabilitative ultrasound imaging (RUSI) serves as a visual biofeedback tool, providing real-time imaging to enhance PFM training, motor learning, and treatment adherence. *Aim*: This narrative review evaluates the role and efficacy of RUSI in pelvic floor rehabilitation. *Method*: A comprehensive search of PubMed, Cochrane, and MEDLINE was conducted using keywords related to pelvic floor rehabilitation, ultrasound, and biofeedback, limited to English-language publications up to July 2025. Systematic reviews, meta-analyses, and clinical trials were prioritized. *Results*: Transperineal and transabdominal ultrasound improve PFM function across diverse populations. In post-prostatectomy men, transperineal ultrasound-guided training enhanced PFM contraction and reduced urinary leakage. In postpartum women with pelvic girdle pain, transabdominal ultrasound-guided biofeedback combined with exercises decreased pain and improved function. Ultrasound-guided pelvic floor muscle contraction demonstrated superior performance compared to verbal instruction. Notably, 57% of participants who were unable to contract the pelvic floor muscles with verbal cues achieved a correct contraction with ultrasound biofeedback, and this approach also resulted in more sustained improvements in PFM strength. Compared to other biofeedback modalities, RUSI demonstrated outcomes that are comparable to or superior to those of alternative methods. However, evidence is limited by a lack of standardized protocols and randomized controlled trials comparing RUSI with other modalities. *Conclusions*: RUSI is an effective visual biofeedback tool that enhances outcomes of PFM training in pelvic floor rehabilitation. It supports clinical decision-making and patient engagement, particularly in cases where traditional assessments are challenging. Further research, including the development of standardized protocols and comparative trials, is necessary to optimize the clinical integration of this method and confirm its superiority over other biofeedback methods.

## Simple Summary

Pelvic floor dysfunction can cause urinary incontinence, pain, and difficulty coordinating the pelvic floor muscles. Many people struggle to learn these muscle contractions using verbal instructions alone. Rehabilitative Ultrasound Imaging (RUSI) provides real-time visual feedback that helps patients see how their pelvic floor muscles move, making training easier and more effective. Research shows that RUSI-guided exercises improve muscle control, reduce symptoms, and increase patient confidence across different groups, including postpartum women and men after prostate surgery. Although RUSI is promising, its use is limited by equipment costs, the need for specialized clinician training, and a lack of standardized protocols. More high-quality studies are needed to confirm its advantages over other biofeedback methods and to guide wider clinical adoption.

## 1. Introduction

Pelvic floor dysfunction encompasses a range of conditions affecting both men and women, with a higher prevalence in women due to anatomical and physiological factors such as pregnancy and childbirth [1]. This disorder manifests primarily in impaired sphincter control, sexual dysfunction, and pelvic pain, which can range from mild discomfort to severe, debilitating symptoms that significantly impact daily activities and quality of life [2]. Symptoms vary widely, necessitating individualized diagnosis and treatment by specialized healthcare professionals. In men, pelvic floor dysfunction is often associated with post-prostatectomy incontinence, while in women, it is frequently linked to urinary incontinence, pelvic organ prolapse, or chronic pelvic pain [3,4].

Pelvic floor muscle (PFM) training is the cornerstone of treatment for stress urinary incontinence (SUI), urge urinary incontinence (UUI), and mixed urinary incontinence (MUI), supported by level A evidence [3,5]. A Cochrane review confirmed that PFM training can cure or reduce symptoms of SUI and other types of urinary incontinence, with 85.8% of women able to correctly activate their pelvic floor muscles in initial assessments following simple verbal instructions [5].

However, 30% to 40% of women struggle to perform a proper voluntary PFM contraction, and up to 70% of those with pelvic floor dysfunction cannot execute contractions correctly, often due to limited proprioceptive awareness [6,7]. This challenge arises from the pelvic floor’s lack of visual feedback and minimal joint movement, which complicate isolating PFM contractions without activating adjacent muscles [8].

The pelvic floor comprises a complex muscular structure extending from the pubic symphysis to the coccyx, with the levator ani muscle group (puborectalis, pubococcygeus, iliococcygeus) providing primary support for pelvic organs and continence control [2]. Optimal PFM function requires coordinated contraction and relaxation patterns that support continence during increased intra-abdominal pressure, facilitate voiding through appropriate relaxation, and maintain structural pelvic organ support [1]. Ghadiri Harati et al. (2024) demonstrated that RUSI can reliably assess these dynamic functions with excellent test–retest reliability (ICC = 0.989) for bladder displacement measurements [9]. Dysfunction occurs when coordination patterns are disrupted, leading to hyperactivity (inability to relax) or hypoactivity (inadequate contraction), which can result in incontinence, pelvic pain, or prolapse symptoms [6].

Biofeedback, used in rehabilitation for over five decades, addresses these challenges by providing real-time biological information to enhance muscle control and treatment adherence [10]. In pelvic floor rehabilitation, biofeedback devices—such as pressure balloons, surface electromyography, or ultrasound imaging—deliver auditory or visual signals that reflect PFM activity [11]. Rehabilitative Ultrasound Imaging (RUSI), as defined in 2006, is a physical therapy tool used to assess muscle and soft-tissue function during dynamic tasks, providing real-time visualization of PFM morphology and movement [12].

By mirroring pelvic floor function to both patients and clinicians, RUSI facilitates accurate PFM retraining, enhances motor learning, and supports the development of personalized treatment protocols [13].

This narrative review aims to evaluate the current applications of RUSI in pelvic floor physical therapy, focusing on its efficacy as a visual biofeedback tool and its impact on treatment outcomes across diverse populations.

Table 1 summarizes the primary clinical applications of RUSI across different patient populations, providing context for the subsequent review of its efficacy as a visual biofeedback tool.

## 2. Methods

A comprehensive literature search was conducted across electronic databases, including PubMed, Cochrane Library, and MEDLINE, to identify studies relevant to pelvic floor health and rehabilitation. The search utilized specific keywords and phrases, such as ‘pelvic floor ultrasound,’ ‘pelvic floor rehabilitation,’ ‘pelvic floor biofeedback,’ ‘biofeedback in rehabilitation,’ ‘visual biofeedback,’ ‘pelvic floor muscle training,’ ‘ultrasound and pelvic floor dysfunction,’ ‘rehabilitative ultrasound and motor control,’ ‘RUSI and motor control,’ ‘transabdominal ultrasound,’ and ‘transperineal ultrasound.’ The search was restricted to English-language publications and extended to July 2025, with no lower date limit to ensure a broad temporal scope.

Studies were selected based on their relevance to RUSI in pelvic floor physical therapy, with a focus on systematic reviews, meta-analyses, and clinical trials. Other study types, such as observational studies and case series, were included if they provided relevant insights into the application or efficacy of RUSI. The selection process involved screening titles and abstracts for relevance, followed by a full-text review to confirm alignment with the aim of the review. Studies were excluded if they focused solely on diagnostic ultrasound without a rehabilitative component or if they were not peer-reviewed. Data extraction focused on the study design, population, intervention, outcomes, and limitations to synthesize evidence on RUSI’s role as a visual biofeedback tool. The narrative synthesis approach was employed to integrate findings, prioritizing high-quality evidence while acknowledging methodological variability across studies.

### 2.1. Overview of RUSI Techniques Used in Included Studies

#### 2.1.1. Transperineal Ultrasound (TPUS)-Guided PFM Training

The patient is positioned in lithotomy or left lateral position. A curved array transducer (2–5 MHz) with acoustic gel is placed on the perineum in the midsagittal plane. Real-time imaging displays the anorectal angle, puborectalis muscle, and anal canal. During contraction, patients observe anterior-superior movement of the anorectal junction and puborectalis thickening. Visual feedback enables correction of inappropriate Valsalva maneuvers or accessory muscle recruitment. Training sessions typically involve 3 sets of 10 contractions with 10-s holds and 10-s rest periods [14].

#### 2.1.2. Transabdominal Ultrasound (TAUS)-Guided Exercises

With the patient supine and the bladder partially filled (150–250 mL), a curved-array transducer (2–5 MHz) is placed suprapubically in the midsagittal plane. The image displays the bladder base, urethra, and posterior bladder wall. During PFM contraction, patients observe bladder base elevation and urethral closure. The clinician provides real-time feedback on contraction quality and teaches discrimination between PFM contraction and abdominal muscle substitution. Exercise protocols typically include phasic contractions (quick flicks) and tonic holds (sustained contractions) guided by visual feedback [15].

#### 2.1.3. TPUS-Guided Manual Therapy

Manual therapy techniques for PFM relaxation are enhanced with TPUS monitoring. The transducer placement follows TPUS-guided training protocols. During manual release techniques applied to the pelvic floor muscles, real-time imaging enables monitoring of tissue compliance changes, modifications in the levator hiatus area, and muscle relaxation patterns. This approach is particularly valuable for treating hyperactive PFM conditions such as chronic pelvic pain or dyspareunia [16].

#### 2.1.4. TAUS/TPUS Biofeedback Delivery

Both approaches provide immediate visual feedback via split-screen displays that show baseline and current muscle activity. Feedback parameters include muscle displacement measurements (in millimeters), contraction duration (in seconds), and relative strength indicators. Audio cues may supplement visual feedback. Treatment progression is documented through measurement standardization and objective tracking of improvement parameters.

## 3. Results

The findings are organized into two subsections: (1) the role of visual biofeedback in motor learning for pelvic floor rehabilitation, and (2) the comparative efficacy of ultrasound-guided training versus other learning methods in pelvic floor disorders. Table 2 and Table 3 summarize key studies and outcomes.

### 3.1. Visual Biofeedback in Motor Learning for Pelvic Floor Rehabilitation

PFMs are critical for pelvic girdle stability, continence, voiding, defecation, sexual function, and childbirth, requiring precise activation for optimal function [2]. Motor learning, driven by neuroplasticity and sensorimotor integration, underpins PFM rehabilitation and involves both implicit and explicit learning processes [22]. Visual biofeedback enhances motor learning by providing real-time cues, improving PFM coordination, strength, and patient motivation [23]. RUSI, including TPUS and TAUS, provides accurate visualization of PFM movement, addressing the limitations of traditional biofeedback devices, such as pressure perineometers, which may misinterpret straining as contraction [24].

A systematic review by Matsi et al. found that visual biofeedback in PFM training improved coordination, response speed, and maximum vaginal pressures, particularly in women with weak PFMs [23]. Biofeedback visualized small contraction amplitudes, boosting patient engagement and adherence. RUSI’s real-time imaging enhances awareness of PFM function and urethral closure, supporting motor learning in conditions like stress urinary incontinence (SUI) and chronic pelvic pain [25,26]. For hyperactive PFM disorders, such as provoked vestibulodynia and endometriosis, RUSI facilitates relaxation training. Del Forno et al. conducted a randomized controlled trial (RCT) with 34 nulliparous women with deep infiltrating endometriosis, using 3D/4D TPUS to assess changes in levator hiatus area after pelvic floor manual therapy [16]. The intervention group showed improved relaxation, reduced pain, and increased therapy adherence, highlighting RUSI’s role in quantifiable feedback. A Cochrane review noted that biofeedback, including RUSI, benefits women unaware of PFM engagement, with TPUS and TAUS being less invasive options [26].

### 3.2. Application and Learning Effects of Ultrasound Compared to Other Learning Methods

#### 3.2.1. Ultrasound-Guided Training Versus PFM Training Without Feedback

TPUS-guided PFM training improves outcomes in post-prostatectomy SUI. Matsunaga et al. conducted a prospective study with 24 men with persistent SUI post-robot-assisted radical prostatectomy (RARP) [14]. After TPUS-guided training (every 2–3 weeks for 3 months), participants showed significant improvements in PFM contraction frequency (7.5 to 10.0 times, *p* < 0.001), sustained contraction duration (2.6 to 9.0 s, *p* = 0.017), urinary leakage reduction (397.0 g to 248.6 g, *p* = 0.024), and quality of life (I-QOL score: 61.0 to 72.1, *p* < 0.001). Despite the lack of a control group, these findings suggest that TPUS enhances motor control and continence [14].

The clinical significance of effective PPUI management extends beyond continence outcomes alone. Rossi et al. demonstrated that post-prostatectomy urinary incontinence independently affects dyadic adjustment and marital relationships (β: −0.25; 95% CI: −4.42, −0.47; *p* = 0.016), underscoring the psychosocial importance of comprehensive rehabilitation approaches, such as RUSI-guided training, that can improve both continence and quality-of-life outcomes [27].

In postpartum women with pelvic girdle pain, TAUS-guided biofeedback combined with stabilization exercises yielded positive outcomes. Kuo et al. conducted a three-arm RCT with 53 women, comparing stabilization exercises alone, exercises with TAUS-guided biofeedback, and a control group. Both exercise groups reduced pain compared to controls (TAUS+exercise: 1.8 vs. 4.4, MD = 2.6, 95% CI [−3.9, −1.2]; exercise only: 2.7 vs. 4.4, MD = 1.7, 95% CI [−3.1, −0.4]) [15]. The TAUS+exercise group showed lower Pelvic Girdle Questionnaire scores (14% vs. 28%, MD = 14, 95% CI [−25, −2]) and faster 6-min walk test times (MD = 0.2, 95% CI [−1.0, −0.2]), indicating superior functional outcomes.

#### 3.2.2. Ultrasound-Guided Training Versus Verbal Instruction

RUSI outperforms verbal instruction in teaching PFM contraction. Dietz et al. evaluated 212 women and found that 26% (n = 56) were unable to perform a correct contraction following verbal instruction [18]. However, 57% (n = 32) of these women achieved a proper contraction after just five minutes of TPUS-guided biofeedback, highlighting its effectiveness in correcting co-contractions and Valsalva maneuvers. Bech et al. conducted a pilot RCT with 31 women over 55 with UI, comparing individualized PFM exercises with and without TAUS guidance [17]. Both groups increased PFM strength at 12 weeks; however, only the TAUS group maintained these gains at 24 weeks (*p* = 0.008), suggesting longer-lasting effects. Yoshida et al. evaluated 116 men post-RARP, of whom 36 received TPUS-guided training [19]. The TPUS group achieved continence faster (75.6 vs. 121.8 days, *p* = 0.037) and had higher 30-day continence rates (52.8% vs. 35.4%, *p* = 0.081), with a hazard ratio of 0.550 (95% CI [0.336, 0.900], *p* = 0.017).

#### 3.2.3. Ultrasound Versus Other Biofeedback Modalities

RUSI shows outcomes comparable to or superior to those of other biofeedback methods (Table 3). Valera-Calero et al. reviewed 11 studies, including one on pelvic floor rehabilitation [20]. They concluded that RUSI enhances motor control with tactile and verbal feedback but shows no significant advantage over pressure biofeedback. Ikeda and Mori conducted an RCT with 65 postpartum women to compare TAUS and vaginal palpation for comprehension of PFM contractions [21]. No significant differences were found in bladder base displacement (TAUS: 0.15 ± 3.28 mm vs. vaginal palpation: 1.11 ± 2.34 mm) or subjective understanding, suggesting both methods are effective, with choice depending on patient preference. No RCTs have directly compared RUSI with intrusive biofeedback modalities like electromyography or manometry in pelvic floor rehabilitation, highlighting a research gap.

### 3.3. Additional Evidence Relevant to RUSI Implementation

Several studies provide supplementary evidence that supports the clinical integration of RUSI and informs its practical application. Ghadiri Harati et al. demonstrated excellent test–retest reliability for transabdominal ultrasound (TAUS) in assessing bladder base displacement, reporting an intraclass correlation coefficient of 0.989 [9]. This finding reinforces the utility of TAUS as a consistent and reproducible method for evaluating pelvic floor muscle activity.

Additional evidence highlights the broader clinical context in which RUSI-guided pelvic floor rehabilitation is implemented. A comprehensive systematic review and meta-analysis by Sacco et al., including 5696 patients across 62 studies, identified several predictors of treatment failure in post-prostatectomy urinary incontinence [28]. These included prior pelvic irradiation (OR: 3.77; 95% CI: 2.27–6.26), higher 24-h pad weight (OR: 1.27; 95% CI: 1.13–1.42), and previous incontinence therapy. These findings underscore the importance of optimizing pelvic floor muscle training strategies—such as incorporating RUSI—to improve outcomes in populations with elevated risk for persistent incontinence.

Together, these studies provide additional context for interpreting the effectiveness and clinical relevance of RUSI across diverse patient groups.

## 4. Discussion

RUSI has emerged as a valuable tool in pelvic floor physical therapy, offering real-time visualization of PFM morphology and function. This section synthesizes the evidence on RUSI’s clinical applications, practical considerations, and challenges, with a focus on its role as a visual biofeedback tool. It is structured into three subsections: Clinical Efficacy and Applications, Practical Implementation Challenges, and Limitations of Current Evidence.

### 4.1. Clinical Efficacy and Applications

RUSI, encompassing both TAUS and TPUS, has demonstrated substantial clinical value in pelvic floor rehabilitation by providing real-time visualization of muscle morphology and function. As outlined in the Section 3, TAUS shows excellent test–retest reliability for bladder base displacement [9], supporting its use as a consistent indicator of pelvic floor muscle activity. These measurement properties strengthen the rationale for incorporating RUSI into clinical decision-making, particularly when precise evaluation of contraction or relaxation patterns is required.

Across diverse patient populations, RUSI enhances motor learning by enabling patients to observe pelvic floor behavior during dynamic tasks directly. This visual feedback improves the accuracy of muscle activation, reduces compensatory strategies, and increases patient engagement—factors that are especially important for individuals who struggle to perceive or isolate pelvic floor contractions [22,23]. Evidence summarized in the Section 3 demonstrates that RUSI-guided training improves continence outcomes in post-prostatectomy men [14,19], reduces pain and enhances function in postpartum women with pelvic girdle pain [15], and supports relaxation training in individuals with hyperactive pelvic floor disorders, such as endometriosis-related pelvic pain [16].

Contextual findings from broader continence research further underscore the clinical relevance of RUSI. As noted in the Results, several patient-related factors—such as prior pelvic irradiation or higher baseline incontinence severity—are associated with poorer rehabilitation outcomes [28]. These insights underscore the importance of optimizing pelvic floor muscle training strategies in high-risk groups, and RUSI may serve as a valuable adjunct to enhance treatment precision and adherence in these populations.

Collectively, the evidence indicates that RUSI supports both assessment and intervention by enhancing motor learning, improving patient comprehension, and enabling individualized treatment progression. Its non-invasive nature and ability to visualize deep musculature make it particularly advantageous in clinical scenarios where internal assessment is not feasible or where patients benefit from enhanced feedback to facilitate correct muscle activation [13,29].

### 4.2. Practical Implementation Challenges

Despite its efficacy, integrating RUSI into clinical practice faces several barriers. First, RUSI requires specialized training, as it is typically absent from entry-level physical therapy curricula, necessitating postgraduate education for safe and competent use. Smith et al. emphasized that point-of-care ultrasound in pelvic health requires specific scope-of-practice guidelines, education frameworks, and governance structures [30]. Current training gaps include a lack of standardized certification processes and variable institutional support for equipment access and education funding. Whittaker et al. established a competency-based educational model for imaging with ultrasound in physical therapy, recommending [13]:

- Didactic training: Foundational knowledge in ultrasound physics, safety protocols, anatomy recognition, and pathology identification.
- Hands-on practice: Supervised clinical sessions with standardized competency assessments.
- Continuing education: Regular updates on technique refinement and evidence-based practice.
- Quality assurance: Ongoing mentorship and peer review of imaging interpretation [13].

Second, RUSI is limited to clinical settings due to equipment requirements, unlike portable biofeedback devices such as electromyography or manometry, which can be used at home [31]. This constraint may reduce accessibility for patients in remote areas or with limited clinical access.

Third, the cost of ultrasound equipment and training can be prohibitive, particularly in resource-constrained settings, though RUSI’s diagnostic and therapeutic versatility may justify its cost-effectiveness over time [13,32]. Equipment costs vary significantly across biofeedback modalities. Alouini et al. noted that while RUSI requires a higher initial investment ($25,000–$60,000 for ultrasound systems), it offers dual diagnostic and therapeutic capabilities [31]. In contrast, EMG biofeedback systems cost $3000–$8000 initially but provide limited diagnostic information. Matsi et al. found that visual biofeedback methods, including RUSI, demonstrated superior patient engagement and treatment adherence compared to verbal instruction alone, potentially reducing overall treatment duration and costs [23]. However, McKiernan et al. noted that RUSI’s equipment requirements limit home-based training, unlike portable EMG devices, which may affect long-term cost-effectiveness by reducing clinic visits [33].

Fourth, patient acceptance of RUSI varies based on multiple factors that influence treatment selection and adherence. Comfort factors include the non-invasive nature of transabdominal approaches versus internal vaginal/anal assessments, with RUSI particularly beneficial for patients with trauma histories or anatomical limitations [13]. Accessibility considerations encompass the clinic-based requirement for RUSI equipment versus portable EMG devices for home training, which potentially limits treatment frequency but provides professional supervision [32]. Treatment adherence factors include enhanced patient engagement through visual feedback, with studies indicating that patients are more motivated when they can observe their muscle function in real-time [23]. However, the need for scheduled clinical sessions may reduce convenience compared to home-based alternatives, requiring individualized assessment of patient priorities and circumstances in treatment selection decisions.

In summary, these implementation challenges—training requirements, equipment limitations, cost considerations, and patient factors—are further complicated by the lack of international standards for RUSI practice in physical therapy. Guidelines exist only as recommendations, lacking consensus on training, competence, and governance [13]. Additionally, delivering effective feedback during RUSI requires alignment with motor learning principles, such as providing knowledge of performance rather than results, which demands clinician expertise [22]. These challenges underscore the need for standardized protocols and accessible training programs to facilitate the integration of RUSI into routine practice.

### 4.3. Limitations of Current Evidence and Review Methodology

This narrative review synthesizes the available literature on RUSI as a visual biofeedback tool in pelvic floor rehabilitation. However, several methodological and evidence-related limitations must be acknowledged.

#### 4.3.1. Limitations of Primary Studies

The evidence supporting RUSI in pelvic floor rehabilitation has notable limitations, summarized in Table 4. Many included studies are characterized by small sample sizes, non-randomized designs, and/or the absence of control groups, which weakens internal validity and limits generalizability [14,17].

There is a notable lack of high-quality randomized controlled trials directly comparing RUSI with other established biofeedback modalities (e.g., surface or intravaginal electromyography, manometry, or pressure perineometry) [20,26].

Measurement reliability, particularly for TPUS, remains a concern due to reported inter-observer variability [32]. Differences in assessor experience and image acquisition techniques may affect outcome consistency.

The evidence base is dominated by short-term outcome assessments, with few studies evaluating the durability of treatment effects beyond 3–6 months [17].

Most investigations have focused on specific populations (e.g., postpartum women, men after robot-assisted radical prostatectomy), leaving limited data on the applicability of RUSI to broader groups, including pediatric patients, older adults, and men or women with other types of pelvic floor dysfunction.

#### 4.3.2. Limitations of the Present Narrative Review

As a narrative rather than a systematic review, the selection of articles was based on relevance rather than predefined systematic criteria, which introduces the potential for selection bias.

No formal quality appraisal tools (e.g., SANRA, AMSTAR, or GRADE) were applied to evaluate individual studies or the overall body of evidence.

The literature search, while comprehensive across major databases (PubMed, Cochrane Library, MEDLINE), may have missed relevant studies published in specialized rehabilitation or allied health journals.

The search was restricted to English-language publications and extended only to July 2025; any relevant studies published after this date are not included.

These limitations collectively indicate that, while RUSI shows promise as an assessment and rehabilitation tool, the current evidence remains preliminary. Firm conclusions regarding the comparative effectiveness, long-term benefits, or optimal implementation of RUSI compared with other biofeedback methods are not yet possible. Well-designed, randomized controlled trials; standardized measurement protocols; and broader population studies are needed to strengthen the evidence base.

### 4.4. Future Directions

Standardized RUSI protocols are essential for clinical consistency. Key needs include standardized technical parameters for TAUS and TPUS, unified training curricula with competency frameworks, evidence-based guidelines for delivering feedback aligned with motor learning principles, consensus outcome measures, and quality assurance methods [13,30]. International professional organizations should develop these standards to facilitate broader adoption while ensuring safety and efficacy [11].

Well-designed randomized controlled trials should compare RUSI with established biofeedback modalities (electromyography, manometry, pressure systems) and evaluate clinical outcomes, cost-effectiveness, and patient preferences [20,26]. Research should expand beyond current populations to include pediatric and elderly patients, with longitudinal studies investigating the durability of long-term benefits [17].

Studies examining implementation barriers across diverse healthcare settings are needed, including cost-effective training models and equipment-sharing strategies [13]. Future developments should explore integrating RUSI with emerging technologies and advancing mobile ultrasound to address portability limitations [33].

These priorities, emphasizing standardization, may establish RUSI’s optimal role in evidence-based pelvic floor rehabilitation while supporting systematic clinical adoption [22].

## 5. Conclusions

Based on available evidence, RUSI appears promising as a visual biofeedback tool in pelvic floor rehabilitation, with preliminary findings suggesting potential benefits for conditions such as stress urinary incontinence, pelvic girdle pain, and chronic pelvic pain. The real-time visualization of PFM function that RUSI provides may enhance motor learning and patient engagement, particularly for individuals who experience challenges with traditional assessments due to pain or anatomical limitations. Compared with verbal instruction, RUSI appears to facilitate more accurate PFM contractions and may offer outcomes comparable to, or potentially superior to, those of other biofeedback methods, such as vaginal palpation, though definitive conclusions require further investigation. Several factors could facilitate RUSI’s broader integration into clinical practice. Clinicians might benefit from specialized training, such as postgraduate programs, to develop proficient application skills. The development of standardized protocols aligned with motor learning principles could potentially improve consistency and effectiveness across clinical settings. Addressing equipment cost barriers through strategies such as shared institutional resources may help enhance accessibility, particularly in resource-limited environments. Future research priorities should include well-designed randomized controlled trials to more definitively compare RUSI with established biofeedback modalities and investigate its long-term efficacy across diverse populations, including pediatric and elderly patients, where current evidence remains limited. Establishing international training and practice standards could provide essential support for the broader clinical adoption of these standards, while ensuring patient safety and treatment quality. Within the limitations of the existing literature, these research and implementation efforts may help better define RUSI’s optimal role in pelvic floor physical therapy and support its judicious clinical application, with the potential to improve patient outcomes when appropriately implemented.

## Figures and Tables

**Table 1:** Clinical Applications of RUSI in Pelvic Floor Rehabilitation.

Population	Condition	RUSI Application	Key Benefits
Post-prostatectomy men	SUI	TPUS-guided PFM training	Improved continence, reduced leakage [14]
Postpartum women	Pelvic girdle pain	TAUS-guided exercises	Reduced pain, enhanced function [15]
Women with endometriosis	Chronic pelvic pain	TPUS-guided manual therapy	Improved relaxation, reduced dyspareunia [16]
Nulliparous women	UI	TAUS/TPUS biofeedback	Enhanced motor learning, sustained PFM strength [17]

**Table 2:** Summary of Key Studies on Ultrasound-Guided PFM Training Outcomes.

Study	Population	Intervention	Key Outcomes	Limitations
Matsunaga et al. [14]	24 men with post-RARP SUI	TPUS-guided PFM training (3 months)	Improved PFM contraction frequency (*p* < 0.001), duration (*p* = 0.017), reduced leakage (*p* = 0.024), better QoL (*p* < 0.001)	No control group, small sample
Kuo et al. [15]	53 postpartum women with pelvic girdle pain	TAUS-guided exercises vs. exercises alone vs. control	Reduced pain (MD = 2.6), lower PGQ scores (MD = 14), faster walk test (MD = 0.2) in TAUS group	No long-term follow-up
Dietz et al. [18]	212 women with UI or post-colposuspension	TPUS-guided biofeedback vs. verbal instruction	57% of verbal instruction failures succeeded with TPUS	Non-randomized, short intervention
Bech et al. [17]	31 women >55 with UI	TAUS-guided PFM exercises vs. exercises alone	TAUS group maintained PFM strength at 24 weeks (*p* = 0.008)	Small sample, pilot study
Yoshida et al. [19]	116 men post-RARP	TPUS-guided PFM training vs. verbal instruction	Faster continence recovery (75.6 vs. 121.8 days, *p* = 0.037)	Non-randomized allocation

**Table 3:** Comparison of RUSI with Other Biofeedback Modalities.

Study	Population	Comparison	Key Outcomes	Limitations
Valera-Calero et al. [20]	Mixed, one pelvic floor study	RUSI vs. tactile/verbal/pressure biofeedback	RUSI is superior to tactile/verbal, comparable to pressure biofeedback	Limited pelvic floor studies
Ikeda & Mori [21]	65 postpartum women	TAUS vs. vaginal palpation	No significant difference in bladder displacement or comprehension	Small effect sizes, short intervention

**Table 4:** Limitations of Current Evidence on RUSI in Pelvic Floor Rehabilitation.

Limitation	Description	Implications
Methodological rigor	Small samples, non-randomized designs, no control groups (e.g., Matsunaga et al. [14])	Limits the strength of evidence, reduces generalizability
Lack of comparative RCTs	No RCTs comparing RUSI with electromyography or manometry (Herderschee et al. [26])	Hinders conclusions on relative efficacy
Reliability variability	Inter-observer variability in TPUS measurements(Roll et al. [32])	Requires standardized protocols, trained assessors
Limited population scope	Focus on specific groups (e.g., postpartum women, men post-RARP)	May overlook broader applications
Short-term focus	Few studies on long-term outcomes (Bech et al. [17])	Uncertainty about sustained benefits

## Data Availability

No new data were created or analyzed in this study.
